# The Effect of CH on Improving the Carbonation Resistance of OPC-CSA Binary Blends

**DOI:** 10.3390/ma16093595

**Published:** 2023-05-08

**Authors:** Shunqin Yang, Guoxin Li, Ge Zhang

**Affiliations:** College of Material Science and Technology, Xi’an University of Architecture & Technology, Xi’an 710055, China

**Keywords:** OPC-CSA binary blends, calcium hydroxide, carbonation resistance, pH value, microstructure

## Abstract

Due to the large amount of CO_2_ generated during steelmaking, to resume production as soon as possible, a fast repair material with good carbonation resistance is needed to repair the factory building. First, the performance of an ordinary Portland cement (OPC)-calcium sulfoaluminate cement (CSA) system under an accelerated carbonization environment was studied. Next, the OPC-CSA system with a CSA content of 15 wt% was selected to be modified by adding calcium hydroxide (CH). The findings showed that the addition of 15 wt% CSA to the OPC-CSA system resulted in the highest mechanical properties. Specifically, the flexural strength and compressive strength after 84 d of carbonization were 18% and 15% higher, respectively, compared to those of OPC alone. The degradation of the mechanical properties of the OPC-CSA system due to carbonation was improved by adding CH. The flexural strength (3.0 wt% CH) and the compressive strength (4.5 wt% CH) of the OPC-CSA-CH system after 84 d of carbonization were 13% and 5% higher, respectively, than those of the OPC-CSA system. The addition of CH increased the alkalinity of the OPC-CSA system and enhanced the stability of Aft, resulting in better carbonation resistance in the OPC-CSA-CH system.

## 1. Introduction

Heavy industry has been playing an important role in the path of industrialization, which has a significant impact on economic growth, economic development, employment, and the environment [1,2]. The iron and steel industry is the basic industry in heavy industry, which has a huge role in promoting economic growth and plays an important role in the national economy [3]. These industries produced a large amount of CO_2_ with the extension of service time, which made it easy to reduce the durability of concrete constructions [4]. In an environment with a high concentration of CO_2_, the main hydration products in concrete, such as AFt, calcium hydroxide (CH) crystals, and calcium silicate hydrate (C-S-H) gel, can easily react with CO_2_, leading to surface cracking [5,6,7]. Therefore, concrete buildings for these purposes were more likely to be damaged by carbonation and to be repaired.

The materials used for quick repair included Portland cement (PC), calcium sulfoaluminate cement (CSA), calcium aluminate cement (CAC), magnesium phosphate cement (MPC), etc. [8,9]. CSA had the advantages of fast setting and hardening, high early strength, micro-expansion, anti-permeability, and anti-freezing properties [10,11,12,13]. Therefore, it had great potential in the application of repair and reinforcement [14]. However, in environments with high CO_2_ concentrations, CSA was more likely to be carbonized. Ye’elimite (C_4_A_3_Ŝ) and C_2_S were the main mineral components of the CSA [15,16,17]. The hydration products of CSA were mainly AFt, AH_3_, and less CH (Equations (1) and (2)) [18]. However, on the one hand, the content of CH was low due to the small content of the silica phase, and on the other hand, CH was more likely to be consumed during hydration, as shown in Equation (3) [19,20,21]. The alkalinity of CSA was low, and it had poor resistance to carbonation. Thus, the amount of CH content was the key factor affecting the alkalinity of concrete and its carbonation resistance.

C_4_A_3_Ŝ + 2CŜH_2_ + 34H = C_6_AŜ_3_H_32_ + 2AH_3_ (1)

2C_2_S + 4H = C_3_S_2_H_3_ + CH (2)

AH_3_ + 3CH + 3CŜH_2_ + 20H = C_6_AŜ_3_H_32_ (3)

Studies showed that the combination of ordinary Portland cement (OPC) and CSA could not only obtain better mechanical properties but also improve the pH value of the CSA system [22]. The main mineral components in OPC were C_3_S, C_2_S, C_3_A, and C_4_AF. When the system was hydrated (Equations (4)–(6)), the high content of the silicon phase led to the formation of a high amount of CH and an increase in alkalinity [23,24,25,26]. Generally, when CSA content was lower than 20 wt% in the OPC-CSA system, the hardening rate of the OPC-CSA system was comparable with that of OPC [27]. If CSA content was higher than 60 wt%, the setting time of OPC-CSA was about 80% shorter than OPC. It was mainly because ye’elimite hydrated more quickly and a lot of AFt crystals formed in the early stage [28,29]. While the hydration of the silica phase in OPC was relatively slow, it provided strength for the OPC-CSA system in the late stage. Therefore, both high early strength and late strength could be achieved for the OPC-CSA system [30,31,32,33,34]. As a result, it had great potential in the field of repair construction, such as urban road construction, airport pavement maintenance, and dam emergency repair.

2C_3_S + 6H = C_3_S_2_H_3_ + 3CH (4)

2C_2_S + 4H = C_3_S_2_H_3_ + CH (5)

3CŜH_2_ + C_3_AH_6_ + 20H = C_6_AŜ_3_H_32_ (6)

Some studies proved that the carbonation resistance of concrete could be enhanced by improving its compactness [35,36,37]. The addition of CH effectively increased the compactness of the hardened CSA system by promoting the hydration of CSA, the formation of more crystalline hydrates, and the reduction of the number of pores [38,39]. When the CH content was 5 wt%, the flexural and compressive strengths of CSA mortar at 3 d were increased by 20% and 30%, respectively [40]. When CH in concrete reacted with CO_2_ to generate CaCO_3_ to fill the pores, it was detrimental to the progress of carbonization.

Therefore, this article adopts CH to improve the carbonation resistance of OPC-CSA binary blends. Firstly, the carbonation resistance of the OPC-CSA system was studied in this research. Then, the OPC-CSA system with a CSA content of 15 wt% was selected to be modified by adding CH. The addition of CH was expected to promote the hydration of the OPC-CSA system and enhance compactness. Finally, the effect of CH on influencing the carbonation resistance of OPC-CSA binary blends was studied through XRD, TG, and SEM testing.

## 2. Experimental

### 2.1. Raw Materials

The chemical compositions of OPC and CSA are listed in Table 1. The physical properties of OPC and CSA are shown in Table 2 and Table 3, respectively. The specific surface of OPC was 345 m^2^/kg, and that of CSA was 355 m^2^/kg. The purity of CH was 98%. Polycarboxylate superplasticizer, retarder (borax decahydrate), and silica sand were used to prepare mortar specimens.

### 2.2. Mix Proportions

The mixing proportions of the three systems are shown in Table 4. OPC was replaced with 0 wt%, 5 wt%, 10 wt%, 15 wt%, and 20 wt% CSA. The CH content accounted for 1.5 wt%, 3.0 wt%, 4.5 wt%, and 6.0 wt% of the total cementitious material; the water-to-binder ratio was 0.28; and the binder-to-sand ratio was 1.0. When the mortar was obtained, these specimens were placed in a standard curing chamber.

### 2.3. Mixing Regime

After dry-mixing the binder for 1 min, water, polycarboxylate superplasticizer, and sand were added to the mixture and then further mixed for 2 min. Secondly, the mixed mortars were cast into the molds, which vibrated for 2 min. Mortars were demolded after 1 h.

### 2.4. Casing and Curing

The specimens cured for 3 d were placed in a standard curing chamber with a temperature of 20 ± 2 °C and a relative humidity of 90% ± 5% for 1 d and then dried in a constant temperature drying oven at 40 °C for 2 d. The specimens were cured for 7 d and then placed in a standard curing chamber with a temperature of 20 ± 2 °C and a relative humidity of 90% ± 5% for an additional 5 d. Afterward, they were dried in a constant-temperature drying oven at 40 °C for 2 d. Finally, the specimens were vertically placed in an accelerated carbon chamber with a temperature of 20 ± 2 °C, a relative humidity of 70% ± 5%, and a CO_2_ concentration of 20% ± 3%.

### 2.5. Experimental Program

#### 2.5.1. Mechanical Strength

The mechanical strength of mortar specimens (40 mm × 40 mm × 160 mm) was tested according to ISO 679:2009. The mortar specimens, cured for 1 and 5 days, were dried in a constant temperature drying oven at 40 °C for 2 d. Then the specimens were vertically placed in an accelerated carbonation chamber. The mechanical strength of the specimens was tested after carbonation for 7 d, 28 d, 56 d, and 84 d, respectively.

#### 2.5.2. Carbonation Depth Test

**Figure 1:**
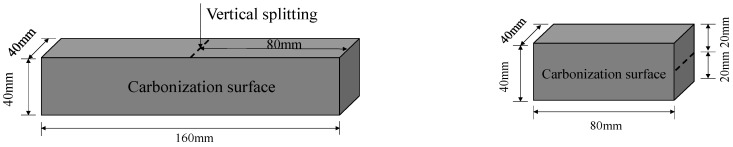
Carbonation depth test.

The specimen was sealed with paraffin wax except for one side for carbonation. The specimen was vertically split from the carbonized surface (as shown in Figure 1). The 1.0% ethanol phenolphthalein solution was sprayed on the new section of the specimen. After 30 s, the carbonation depth was measured with an electronic display vernier caliper. Three points were measured on each side of the cross-section. The resulting average was accurate to 0.01 mm.

#### 2.5.3. pH Value Test

The *w*/*c* ratio of pastes used for pH value testing was 10.0. The water used in this work was deionized water. The pH value of the pastes was measured by the pH meter. The pH of OPC-CSA and OPC-CSA-CH mortar specimens were tested, respectively.

#### 2.5.4. XRD Analysis

The hydration products of the samples were tested by X-ray diffraction (XRD). For XRD (Model D/max-3c, Rikagu, Tokyo, Japan) analysis, samples were terminated by hydration with anhydrous ethanol. A Cu Kα target with a scanning range of 5° to 50° (2θ) was used.

#### 2.5.5. Thermal Analysis (TG/DTA)

A SDTQ 600 TA thermogravimetric analyzer was used. The testing temperature range was from 25 to 800 °C. The heating rate was 10 °C/min, and the protective gas was N_2_.

#### 2.5.6. Morphology

The morphology of samples was observed using scanning electron microscopy (SEM). Samples for SEM (Gemini SEM 500, Zeiss, Oberkochen, Germany) analysis were cut into small pieces and hydrated with anhydrous ethanol. Before testing, a layer of Au was sprayed to increase electrical conductivity.

## 3. Results and Discussion

### 3.1. The Properties of OPC-CSA Binary Blends

#### 3.1.1. Flexural and Compressive Strength

**Figure 2:**
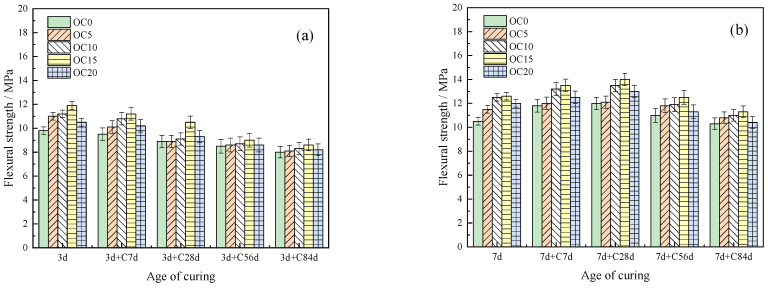
Flexural strength of the OPC-CSA system at different curing ages: (**a**) 3 d and (**b**) 7 d.

The influence of CSA content and curing time on the flexural strength of the OPC-CSA system after carbonation is shown in Figure 2a,b. From Figure 2a, the flexural strength of the OPC and OPC-CSA systems decreased with the increase in carbonation age. Additionally, the flexural strength was increased by adding CSA at each age. Moreover, it first increased and then decreased with the increasing CSA in the OPC-CSA system. When the CSA content was 15 wt%, the flexural strength was the highest, which was 8.9 MPa at 84 d. From Figure 2b, when the curing time was prolonged to 7 d, the flexural strength of the OPC and OPC-CSA systems first increased until 28 d and then decreased under the carbonation environment. The hydration of the OPC-CSA system was promoted with the increase in time and the addition of CSA. The content of CH, C-S-H, and AFt was increased, and the porosity was reduced [41]. On the one hand, as CO_2_ entered the interior, it reacted with CH crystal and C-S-H gel to produce CaCO_3_. This caused the surface to become brittle and hard, resulting in a reduction in flexural strength. On the other hand, as the carbonation time increased and the amount of CSA added increased, the carbonation-resistant substance in the concrete increased, improving its carbonation resistance [42]. The porosity was reduced, and the decline in flexural strength was delayed.

**Figure 3:**
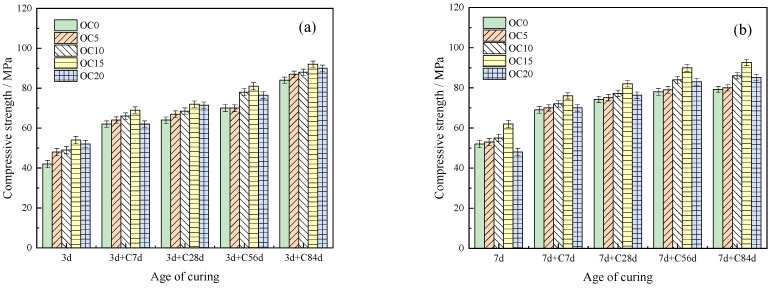
Compressive strength of OPC-CSA binary blends at different curing ages: (**a**) 3 d and (**b**) 7 d.

The influence of CSA content and curing time on the compressive strength of the OPC-CSA system after carbonation is shown in Figure 3a,b. The compressive strength of the OPC and OPC-CSA systems increased with the prolongation of the carbonation age. The addition of CSA increased the compressive strength at each age. When the CSA content was 15 wt%, the compressive strength reached its maximum. Compared to Figure 3a, the compressive strength of the OPC-CSA system significantly increased when the curing time was extended from 3 d to 7 d, as shown in Figure 3b. Due to the increase in time, the crystals in the matrix were more cross-linked with each other. The AFt content was increased with the addition of CSA. Therefore, the spatial network structure of the system became denser. In addition, with the process of carbonization, a large amount of CaCO_3_ was added to the OPC-CSA system. Thus, the pores were reduced in the system, and the compressive strength was improved [43].

#### 3.1.2. Carbonation Depth

The carbonation depth of the OPC-CSA system after being cured for 3 and 7 days was tested, respectively, as shown in Table 5. From Table 5, the carbonation depth of the OPC-CSA system increased with increasing CSA content. It could be concluded that the content of the formed CH was reduced due to the replacement of CSA with OPC. In addition, the formation of AFt, which is the main hydration product of CSA, consumed CH, thus further reducing the alkalinity and carbonation resistance of the system. Moreover, as the curing time increased from 3 d to 7 d, the carbonation depth of the OPC-CSA system was further reduced, and the generation time of the carbonation layer was longer. This is mainly attributed to the fact that the number of hydration products increased significantly during the prolonged curing period. The denser internal structure hindered the infiltration of CO_2_.

Although the OPC-CSA system had higher flexural strength and compressive strength than OPC in a carbonation environment, its carbonation depth was deeper than OPC. With the increase in CSA content, the carbonation depth tends to increase. To further improve the carbonation resistance of the OPC-CSA system, CH was added to form the CPC-CSA-CH system.

### 3.2. The Properties of OPC-CSA-CH Ternary Blends

#### 3.2.1. Flexural and Compressive Strength after Carbonation

**Figure 4:**
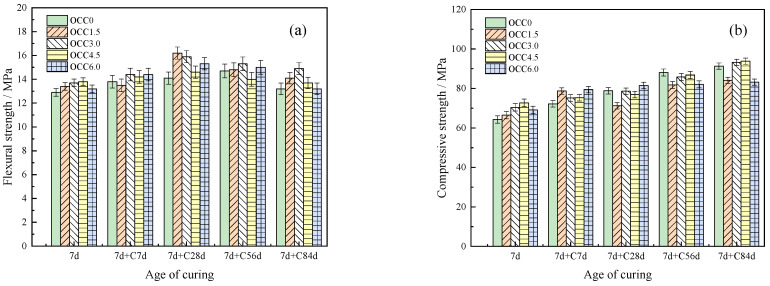
Flexural and compressive strength of OPC-CSA-CH ternary blends after carbonation. (**a**) Flexural strength and (**b**) compressive strength.

The flexural strength and compressive strength of the OPC-CSA-CH system after carbonation are shown in Figure 4a,b, respectively. The flexural strength of the OPC-CSA-CH system with different CH content first increased until 28 d and then decreased with the increasing carbonation age. As carbonation progresses, the formation of CaCO_3_ and the decomposition of C-S-H gels lead to a brittle and hard matrix. Therefore, the flexural strength decreases. The addition of CH effectively enhanced the flexural strength of the OPC-CSA system. When the CH content was 3.0 wt%, the flexural strength of the OPC-CSA-CH system reached its maximum.

From Figure 4b, the compressive strength of the OPC-CSA-CH system increased with the increasing carbonation age. When the CH content was 4.5 wt%, the compressive strength of the OPC-CSA-CH system could reach its maximum, which was 92.6 MPa at 84 d. Therefore, an appropriate dosage of CH was beneficial to improve the strength of the OPC-CSA system in the carbonation environment.

It could be speculated that the addition of an appropriate amount of CH increased the alkalinity of the system, promoted the early hydration of CSA, and led to an increase in AFt and CH content. On the one hand, the system’s early mechanical properties were improved with an increase in AFt. On the other hand, the alkaline reserve increased with the addition of CH [44]. When CH on the surface reacted with CO_2_ to generate CaCO_3_, the channels for CO_2_ to enter the interior were relatively reduced, which was unfavorable for carbonization. Therefore, an appropriate amount of CH improved the mechanical properties of the OPC-CSA system after carbonization. However, excessive CH content could lead to excessive Ca^2+^ concentration, and the dissolution rate of C_3_S was reduced. Replacing part of the cement with CH, resulted in a lower AFt content, which was unfavorable for improving the mechanical properties after carbonization [45].

#### 3.2.2. pH Value Development

**Figure 5:**
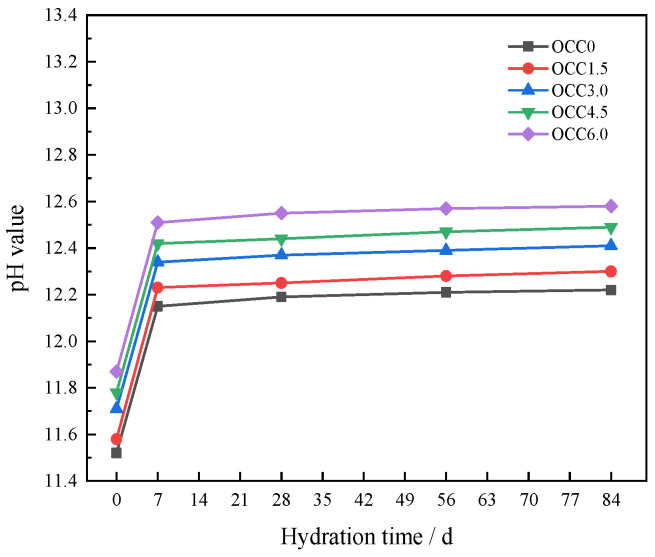
Development of the pH value of OPC-CSA-CH ternary blends.

Figure 5 shows the effect of CH on the pH value of the OPC-CSA-CH system. From Figure 5, after 7 d of hydration, the pH value of the OPC-CSA-CH system significantly increased. However, the pH value began to slowly increase after 7 d. At the same age, the pH value increased with the CH content. The alkalinity of OCC3.0 was observed to have a significant improvement compared to OCC0 and OCC1.5. However, the alkalinity of OCC6.0 did not show a significant improvement compared to OCC3.0 and OCC4.5. It could be speculated that when the CH content in the OPC-CSA-CH system exceeded 3.0 wt%, the CH content gradually saturated and the pH growth rate decreased. When the content of CH in the OPC-CSA system was 6.0 wt%, the pH value of the OPC-CSA-CH system reached 12.5 at 84 d, which was higher than that of the OPC-CSA system with a pH value of only 12.1. This phenomenon proved that the alkaline reserve of the OPC-CSA system was improved by adding CH. C_4_A_3_Ŝ primarily generated AFt in the early hydration of CSA. In the C_4_A_3_Ŝ phase, Al^3+^ existed as an Al-O tetrahedron, while in the AFt phase, Al^3+^ existed as an Al-O octahedron. The formation of AFt by the liquid-phase reaction was the result of the conversion process from four coordinated Al^3+^ to six coordinated Al^3+^. This process could be promoted by an alkaline environment. The alkalinity of the CSA hydration environment was increased by adding CH, reducing the free energy of nucleation of the Al-O octahedron and the critical size of the crystal nucleus. The formation rate of Al-O octahedral crystal nuclei was increased, thus promoting the hydration of CSA [46]. On the one hand, CH improved the hydration of the OPC-CSA system. On the other hand, the CH in the solid phase became less soluble as the alkali reserve increased. The OPC-CSA-CH system was relatively stable internally, which was beneficial for improving carbonation resistance.

#### 3.2.3. Carbonation Depth

Table 6 shows the effects of CH content on the carbonation depth of the OPC-CSA system. From Table 6, the addition of CH effectively decreased the carbonation depth at each age. With the increasing CH content, the carbonation depth continuously decreased, indicating better carbonation resistance. On the one hand, the carbonation products produced on the surface of the samples hindered the further infiltration of CO_2_. On the other hand, the increased alkalinity hindered the dissolution of CH, leading to a slowing down of the rate of the carbonation reaction. Therefore, the more CH content in the OPC-CSA-CH system, the lower the carbonation depth.

#### 3.2.4. X-ray Diffraction Analysis (XRD)

**Figure 6:**
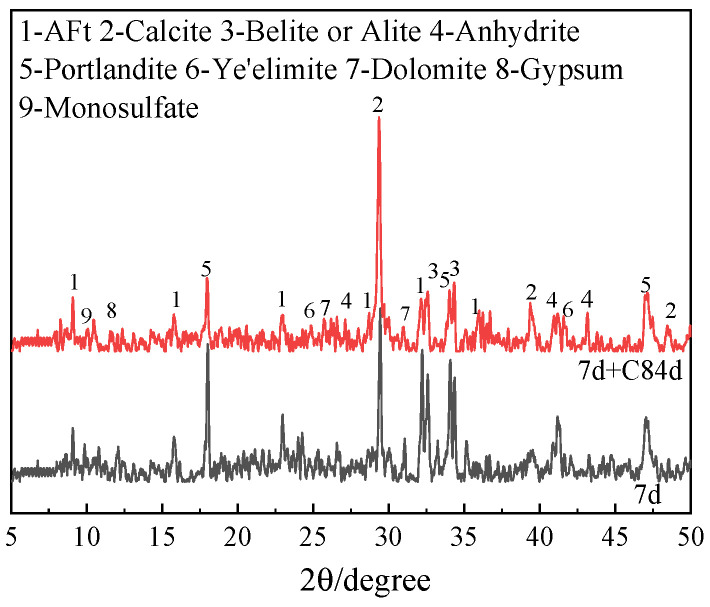
XRD patterns of OCC0.

**Figure 7:**
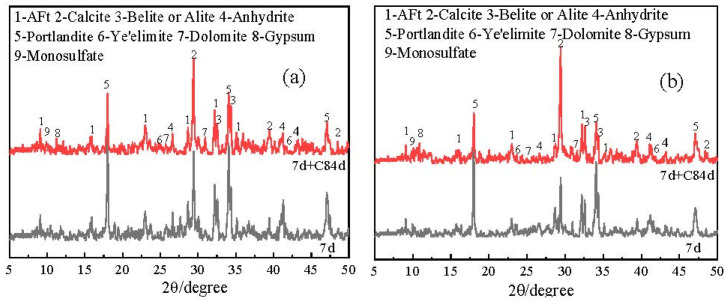
XRD patterns of OPC-CSA-CH ternary blends. (**a**) OCC3.0; (**b**) OCC6.0.

XRD patterns of the OPC-CSA-CH system added with 0 wt%, 3.0 wt%, and 6.0 wt% CH are shown in Figure 6 and Figure 7. From Figure 6 and Figure 7, the main crystal hydration products of the OPC-CSA system were not changed by the addition of CH; they were AFt, CH, and less monosulfate. In addition, as the carbonation age increased, the intensity of the CaCO_3_ XRD patterns significantly increased while that of CH decreased markedly. This indicates that the CH had transformed into CaCO_3_ in the carbonation environment. Moreover, the intensity of the AFt patterns was increased by the addition of CH. On the one hand, the presence of CH could promote an increase in hydration rate and improve the degree of hydration in the OPC-CSA system. On the other hand, the increase in alkalinity could enhance the stability of AFt, thereby increasing the density of the matrix and maintaining the strength of the system [47,48].

#### 3.2.5. Thermal Analysis

**Figure 8:**
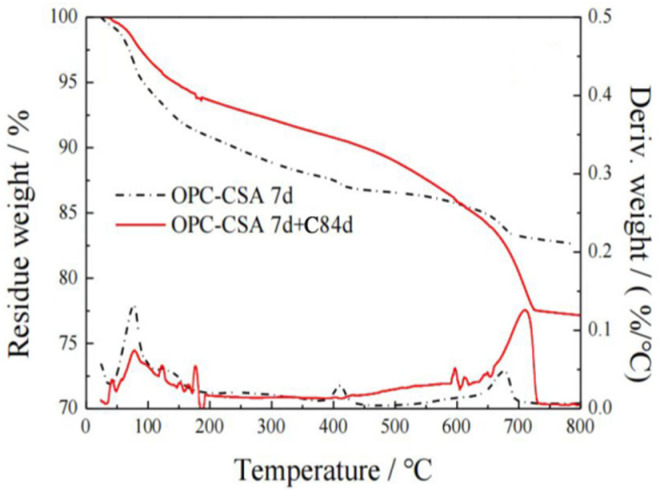
Thermogravimetric curves of OCC0.

**Figure 9:**
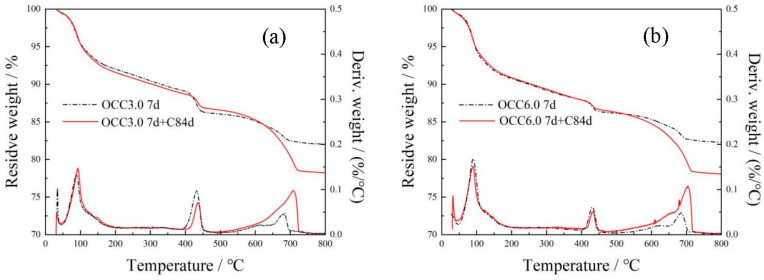
Thermogravimetric curves of OPC-CSA-CH ternary blends. (**a**) OCC3.0; (**b**) OCC6.0.

The TG/DTA curves of the OPC-CSA-CH system added with 0 wt%, 3.0 wt%, and 6.0 wt% CH are shown in Figure 8 and Figure 9. Three different weight loss peaks at 50–150 °C, 380–450 °C, and 600–750 °C appeared in the DTA curves. In the temperature range of 50–150 °C, crystal water in AFt was removed, leading to a decrease in mass. Thus, the content of AFt could be calculated according to Equation (7). In the temperature range of 380–450 °C, the dehydration of Ca(OH)_2_ led to a decrease in mass (Equation (8)). The content of Ca(OH)_2_ could be calculated according to Equation (9). In the temperature range of 600–750 °C, the CaCO3 decomposed (Equation (10)), and its content could be calculated according to Equation (11). The results of the content of AFt, Ca(OH)_2_, and CaCO3 are listed in Table 7.

(7) $$\mathrm{AFt}(\%)=\left(\frac{M_{50}-M_{150}}{M_{\mathrm{total}}}\right)/0.35$$

Ca(OH)_2_ = CaO + H_2_O (8)

(9) $$\mathrm{Ca}(\mathrm{OH}{)}_{2}\;(\%)=\frac{M_{\mathrm{CH}}\times \left(M_{380}-M_{450}\right)}{M_{H_{2}O}\times M_{\mathrm{total}}}$$

where M_CH_ is the molar mass of Ca(OH)_2_, g/mol, and MH2O is the molar mass of H_2_O, g/mol.

CaCO_3_ = CaO + CO_2_ (10)

(11) $${\mathrm{CaCO}}_{3}\;(\%)=\frac{M_{{\mathrm{CaCO}}_{3}}\times \left(M_{600}-M_{750}\right)}{M_{{\mathrm{CO}}_{2}}\times M_{\mathrm{total}}}$$

where M_CaCO_3__ is the molar mass of CaCO_3_, g/mol, and M_CO_2__ is the molar mass of CO_2_, g/mol.

From Table 7, the presence of CH significantly increased the amount of AFt at 7 d before carbonation, indicating that the hydration was accelerated by CH. After 84 d carbonation, the OPC-CSA system without CH showed a significant reduction in AFt content from 19.83% to 13.51%, while the OPC-CSA-CH system, which was added with 3.0 wt% CH, only exhibited a slight decrease in AFt content from 22.98% to 22.31%. It could be concluded that the higher alkalinity was beneficial to the stability of AFt during carbonation. Additionally, when the content of CH further increased from 3.0 wt% to 6.0 wt%, the increase in AFt content was small.

Moreover, the amount of CH in hydration products was also significantly increased by the addition of CH. With the additional CH, the amount of CH in hydration products continuously increased. After 84 d carbonation, the content of CH was as high as 8.98% for OCC6.0, which was comparable to that of the OPC-CSA system before carbonation. Simultaneously, it could be noticed that the content of CaCO3 sharply decreased with increasing CH. This indicates that the OPC-CSA-CH system with higher additional CH content had better carbonation resistance. This was consistent with the carbonation depth results [49].

#### 3.2.6. Morphology

**Figure 10:**
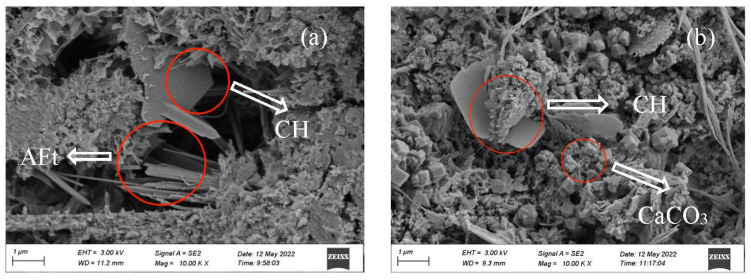
SEM images of OCC0: (**a**) 7 d and (**b**) 7 d + C84d.

**Figure 11:**
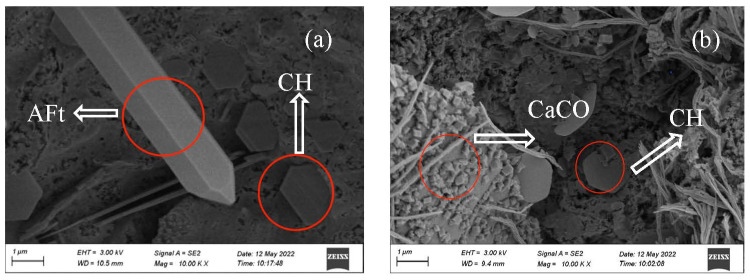
SEM images of OCC3.0: (**a**) 7 d and (**b**) 7 d + C84d.

**Figure 12:**
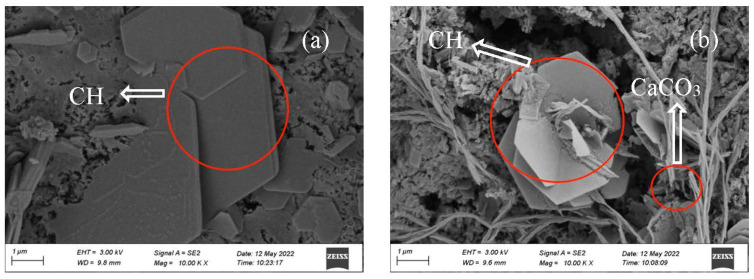
SEM images of OCC6.0: (**a**) 7 d and (**b**) 7 d + C84d.

The morphology of the OPC-CSA-CH system added with 0 wt%, 3.0 wt%, and 6.0 wt% CH is shown in Figure 10, Figure 11 and Figure 12. From Figure 10a, when no CH was added, there were a large number of needle-like AFt crystals and a small amount of hexagonal plate-shaped CH crystals in the OPC-CSA system at 7 d before carbonation. After 84 d carbonation, the number of micropores in the OPC-CSA system decreased, and there were a few hexagonal plate-shaped CH crystals observed. The degree of CH crystals in Figure 10b was much smaller than that in Figure 10a. This indicates that the CH had been consumed during carbonation, and simultaneously, the formed CaCO3 filled in pores and compacted the matrix. Thus, the compressive strength was enhanced. In Figure 11a and Figure 12a, a large number of CH crystals and needle-like AFt crystals could be observed. After 84 d carbonation, a significant amount of AFt crystals and certain CH crystals were still present in the OPC-CSA-CH system with 3.0 wt% and 6.0 wt% CH, respectively. Due to the denser matrix, the intrusion of CO_2_ was hindered. Therefore, the carbonation resistance was improved.

## 4. Conclusions

The performance of the OPC-CSA system and the OPC-CSA-CH system under a carbonation environment was investigated in this study. According to the results, the following conclusions can be drawn:

1. Partial replacement of CSA proved to be effective in enhancing the flexural strength and compressive strength of OPC under carbonation environments. The mechanical strength was found to be maximum when the CSA content was 15 wt%. The addition of CH effectively enhanced both the carbonation resistance and mechanical strength of the system. The OPC-CSA-CH system had the highest flexural strength and compressive strength when the CH content was 3.0 wt% and 4.5 wt%, respectively.
2. The carbonation resistance of the OPC-CSA system decreased as the CSA content increased. At a CSA content of 20 wt%, the carbonization depth was 147% higher than that of OPC. In contrast, the carbonation resistance of the OPC-CSA-CH system increased with an increase in CH content. When the CH content was 6.0 wt%, the carbonization depth of the OPC-CSA-CH system was 42.7% lower than that of the OPC-CSA system.
3. The hydration of the OPC-CSA system was promoted with the increase in time and the addition of CSA. The carbonation-resistant substance was increased and the porosity was reduced, which was beneficial to improving the mechanical properties after carbonization.
4. The alkalinity of the OPC-CSA system was increased with the addition of an appropriate amount of CH. Early hydration was promoted, and AFt and CH content in the OPC-CSA system were increased. On the one hand, when the surface CH reacted with CO_2_ to generate CaCO_3_, the channels for CO_2_ to enter the interior were relatively reduced, which was unfavorable for carbonization. On the other hand, the CH in the solid phase became less soluble as the alkali reserve increased. The OPC-CSA-CH system was relatively stable internally, and the stability of AFt was increased. Thus, the OPC-CSA-CH system had better carbonation resistance.
5. The evolution of microstructure is an important aspect that requires further investigation in this study. Therefore, future research will focus on exploring this area to gain a better understanding of the system’s properties and behavior.

## Figures and Tables

**Table 1:** Chemical composition of OPC and CSA.

Components	SiO_2_	Al_2_O_3_	Fe_2_O_3_	CaO	MgO	SO_3_	K_2_O	Na_2_O	Loss
OPC/wt%	22.32	5.84	3.15	61.23	2.02	2.00	0.39	0.15	1.66
CSA/wt%	6.86	36.46	2.28	42.25	1.33	8.82	0.18	0.22	0.67

**Table 2:** Physical properties of OPC.

W/C	S/C	Setting Time/Min		Flexural Strength/MPa		Compressive Strength/MPa	
		Initial	Final	3 d	28 d	3 d	28 d
0.50	3.0	185	221	5.4	8.5	22.8	48.2

**Table 3:** Physical properties of CSA.

W/C	S/C	Setting Time/Min		Flexural Strength/MPa		Compressive Strength/MPa	
		Initial	Final	1 d	7 d	1 d	7 d
0.50	3.0	34	60	5.1	8.5	34.2	45.8

**Table 4:** Mix design of composite.

Number	Weight/g					Superplasticizer/wt%	Retarder/wt%
	OPC	CSA	Ca(OH)_2_	Water	Sand		
OC0	900.0	0	0	252.0	900.0	0.6	0
OC5	855.0	45.0	0	252.0	900.0	0.8	0.5
OC10	810.0	90.0	0	252.0	900.0	0.8	0.5
OC15	765.0	135.0	0	252.0	900.0	0.8	0.5
OC20	720.0	180.0	0	252.0	900.0	0.8	0.5
OCC0	765.0	135.0	0	252.0	900.0	0.8	0.5
OCC1.5	753.5	133.0	13.5	252.0	900.0	0.8	0.5
OCC3.0	742.1	131.0	27.0	252.0	900.0	0.8	0.5
OCC4.5	730.6	128.9	40.5	252.0	900.0	0.8	0.5
OCC6.0	719.1	126.9	54.0	252.0	900.0	0.8	0.5

OC0, OC5, OC10, OC15, and OC20 represented that OPC was replaced with 0 wt%, 5 wt%, 10 wt%, 15 wt%, and 20 wt% CSA, respectively. OCC0, OCC1.5, OCC3.0, OCC4.5, and OCC6.0 represented that CH content accounted for 0 wt%, 1.5 wt%, 3.0 wt%, 4.5 wt%, and 6.0 wt% of the total cementitious material, respectively.

**Table 5:** Carbonation depth of OPC-CSA binary blends after curing/mm.

Age		OC0	OC5	OC10	OC15	OC20
Standard curing for 3 d	+C7d	0.00	1.58	2.21	2.53	2.65
Standard curing for 3 d	+C28d	1.71	2.39	3.21	3.38	3.58
Standard curing for 3 d	+C56d	1.94	3.01	3.56	3.78	3.81
Standard curing for 3 d	+C84d	2.03	3.11	3.60	3.80	3.92
Standard curing for 7 d	+C7d	0.00	0.93	1.58	2.16	2.51
Standard curing for 7 d	+C28d	0.00	1.23	2.11	2.79	3.13
Standard curing for 7 d	+C56d	0.46	1.98	2.56	3.36	3.53
Standard curing for 7 d	+C84d	1.52	2.45	2.76	3.42	3.76

**Table 6:** Carbonation depth of OPC-CSA-CH ternary blends after curing/mm.

Age		OCC0	OCC1.5	OCC3.0	OCC4.5	OCC6.0
Standard curing for 7 d	+C7d	2.16	1.35	0.85	0.50	0
Standard curing for 7 d	+C28d	2.79	2.39	1.62	1.58	1.31
Standard curing for 7 d	+C56d	3.36	2.73	2.06	1.82	1.71
Standard curing for 3 d	+C84d	3.42	2.99	2.33	2.01	1.96

**Table 7:** The evolution of ettringite, calcium hydroxide, and calcium carbonate content in OPC-CSA-CH ternary blends.

System	Age	Content of Products/wt%		
		AFt	Ca(OH)_2_	CaCO_3_
OCC0	S7d	19.83	8.26	6.45
OCC0	S7d + C84d	13.51	4.23	17.20
OCC3.0	S7d	22.98	12.62	6.31
OCC3.0	S7d + C84d	22.31	6.28	14.23
OCC6.0	S7d	23.39	13.81	6.78
OCC6.0	S7d + C84d	22.01	8.98	13.48
